# Quality of life 1 year after hospital discharge in unvaccinated pregnant women with COVID-19 respiratory symptoms: a prospective observational study (ODISSEA-PINK study)

**DOI:** 10.3389/fmed.2023.1225648

**Published:** 2023-09-06

**Authors:** Luigi Vetrugno, Alessia Sala, Cristian Deana, Francesco Meroi, Maria Grandesso, Salvatore Maurizio Maggiore, Miriam Isola, Maria De Martino, Stefano Restaino, Giuseppe Vizzielli, Tiziana Bove, Lorenza Driul

**Affiliations:** ^1^Department of Medical, Oral, and Biotechnological Sciences, University of Chieti-Pescara, Chieti, Italy; ^2^Department of Anesthesiology, Critical Care Medicine and Emergency, SS. Annunziata Hospital, Chieti, Italy; ^3^Department of Medical Area (DAME), University of Udine, Udine, Italy; ^4^Department of Obstetrics and Gynaecology, ASUFC, Ospedale Santa Maria Della Misericordia, Udine, Italy; ^5^Department of Anesthesia and Intensive Care, Health Integrated Agency of Friuli Centrale, Academic Hospital of Udine, Udine, Italy; ^6^Department of Innovative Technologies in Medicine and Dentistry, Gabriele d'Annunzio University of Chieti Pescara, Chieti, Italy

**Keywords:** COVID-19, quality of life, post-traumatic stress disorder, pregnancy, pneumonia

## Abstract

**Introduction:**

Little is known about Quality of Life within the first court of unvaccinated COVID-19 pregnant women exposed to the pandemic stressor. Primary aim of this study was to evaluate 1 year after hospital discharge HRQoL in a cohort of COVID-19 unvaccinated pregnant patients with COVID-19.

**Methods:**

in this prospective observational study, all COVID-19 positive pregnant women at any gestational age, admitted to the Obstetric Department at the University Hospital of Udine, Italy, from 1 March 2020 to 1 March 2021, requiring or not oxygen supplementation due to SARS-CoV2 pneumonia were evaluated. Patients with a history of neurological or psychiatric disease, those with a previous abortion, and those who refused to provide written informed consent were excluded from the study. We investigated pregnant positive COVID-19 women Health-related quality of life (HRQoL) with the Short-Form Health Survey-36 (SF-36) and Post-traumatic Stress-Disorder (PTSD) with the Impact of Event Scale-Revised (IES-R).

**Results:**

62 pregnant women respected the inclusion criteria of the study, and data from 33 patients were analyzed. The mean age was 32 ± 6 years, with a median gestational age of 38 weeks [IQR 34–40]. 15.2% of patients required oxygen therapy through noninvasive respiratory support (with high flow nasal cannula) for a median of 9 days [IQR 6–12]. The median Physical Component Summary (PCS) and Mental Component Summary (MCS) scores were 50.2 [IQR 46.7–53.7] and 56.0 [IQR 46.8–60.6] respectively. Ten patients out of 33 (30%) tested positive for PTSD. Maternal age, gestational age, and history of cardiac-pulmonary-kidney disease significantly affected HRQoL at multivariable analysis.

**Discussion:**

In COVID-19 pregnant unvaccinated women some physical impairments reducing HRQoL are still present 1 year after hospital discharge. Previous medical history such as history of cardiac-pulmonary-kidney disease significantly affected HRQoL. Long and repeated follow-up should be pursued in this category of patients.

**Clinical trial registration:**ClinicalTrials.gov, Identifier NCT04860687.

## Introduction

During the early phase of the SARS-CoV-2 pandemic, pregnant women progressively became one of the affected populations suffering from respiratory COVID-19 interstitial pneumonia (1).

During that period, the first and second waves, a vaccine was not available on the market. Later, some concerns about the vaccine’s safety during pregnancy precluded administering it to this group of patients (2).

In the Americas, more than 365,000 cases of SARS-CoV2 in pregnant women have been identified so far, of which 9% had severe disease and required hospitalization, isolation, non-invasive ventilation outside ICU, with 4% ICU admission and invasive mechanical ventilation in 2.9 per 1,000 cases (3–5).

The increased risk of developing COVID-19 infection and related symptoms is probably due to physiological changes associated with pregnancy, such as increased abdominal volume with decreased respiratory reserve (RFC), immune system adaptation to the newborn, and different comorbidities such as obesity, diabetes, hypertension, and cardiovascular disease (6–8).

At the beginning of 2021, vaccination became possible for pregnant women, and it was endorsed by the national scientific societies, with a decrease in the number of cases and, nowadays, the pandemic expected to end probably by the end of 2023, as recently declared by World Health Organization (WHO) Director Dr. Tedros Adhanom Ghebreyesus (9, 10).

However, little is known about the first group of unvaccinated pregnant women who were subjected to contact restrictions to limit the viral spread and reduced visits from their loved ones during hospital stay. Whether this significantly impacted their health and quality of life remains poorly investigated (11).

Therefore, we performed this study to evaluate the Health-Related Quality of life (HRQoL) after 1 year of discharge in unvaccinated pregnant patients with COVID-19.

Secondary aims included identifying possible correlations between HRQoL 1 year after discharge and demographic, medical, or clinical data and screening for post-traumatic stress disorder (PTSD) as well as detecting risk factors and investigating whether PTSD impacts HRQoL.

## Methods

### Study setting and design

This prospective observational study was conducted at the University Hospital of Udine, Italy. The regional Ethics Committee of Friuli-Venezia Giulia (CEUR) approved the study on 15 February 2022, designating it with the number CEUR-2022-Em-58. The study was registered in ClinicalTrials.gov with the number NCT04860687. Before enrollment in the study, all patients signed written informed consent.

### Patients’ characteristics

We investigated pregnant women at any gestational age with positive COVID-19 assay from either nasal or pharyngeal swabs who were admitted to the Department of Obstetrics and Gynecology at the Academic Hospital of Udine with a PaO_2_/F_I_O_2_ ratio ≤ 300 requiring or not requiring oxygen supplementation (F_I_O_2_ ≥ 21%) under conventional oxygen therapy (COT), high flow nasal oxygen (HFNO), continuous positive airway pressure (CPAP), or pressure support ventilation (PSV). Exclusion criteria were patients with a history of neurological or psychiatric disease, those with a previous abortion, and those who refused to provide written informed consent.

Two investigators (CD and AS) were responsible for screening and patients’ inclusion, specifically considering a 1-year follow-up after hospital discharge. The investigators contacted the patients by phone and sent the patients’ self-reported questionnaires by email.

We considered “lost at the follow-up” patients who did not answer after three phone calls and did not return the completed questionnaires 4 weeks after receiving them.

We investigated HRQoL with the Short-Form Health Survey-36 (SF-36), while the Impact of Event Scale-Revised (IES-R) was used to investigate post-traumatic stress-disorder (PTSD).

The SF-36 patient-reported questionnaire incorporates 36 items that evaluate the HRQoL and produces 8 scaled scores. Every scale is transformed into a 0–100 value, with each question carrying equal weight. A higher score is related to better quality of life and vice versa.

This questionnaire is divided into specific domains: (i) physical functioning (PF), which reflects the extent to which general health limits physical activity; (ii) physical role (PR), which expresses how physical health interferes with work or limits activity; (iii) bodily pain (BP), which analyzes the intensity of pain and the effect of pain on a patient’s ability to work; (iv) general health (GH), a patient’s evaluation of their health or health outlook; (v) vitality (VT), which includes the energy the patient has; (vi) social functioning (SF), a measure of how health or emotional problems interfere with social activities; (vii) emotional role (RE), an evaluation of the extent to which emotional problems interfere with work or activities; and (viii) mental health (MH), a global assessment of general mental health.

The eight SF-36 domains can be collapsed to create two global components: the physical component summary (PCS) and the mental component summary (MCS), according to the method proposed by Ware et al. (12).

The PCS is principally derived from PF, PR, and BP, while for the MCS, the major determinants are MH, RE, and SF. VT and GH are equally determinants of both summary scores.

In practice, the PCS reflects physical wellness, while MCS returns information on the global mental health condition. They are constructed using a principal component analysis based on the data of the general population of the US, standardized to obtain a mean of 50 and a standard deviation of 10.

IES-R is a self-reported scale, with items rated on a 5-point Likert scale ranging from 0 to 4. A 22-item questionnaire measures subjective distress caused by traumatic events. The total score ranges from 0 (minimum) to 88 (maximum).

Three subscale scores define intrusion, avoidance, and hyperarousal aspects of PTSD. Sum scores equal to or greater than 33 indicate the probable presence of PTSD (13).

### Data collection

We collected demographic data such as age, gender, gestational age, weight at hospital admission and follow-up, level of education (none, compulsory school, high school degree, or college degree), marital status (single, married, separated/divorced, or widowed), cohabitation (yes or no), employment (unemployed or active worker), previous medical history, hospital admission date for COVID-19, and discharge date. Clinical data included oxygen therapy, type of oxygen support, duration, and steroid use.

### Statistical analysis

Categorical variables were presented as absolute values (percentages), and continuous variables as medians and interquartile ranges [IQR] or mean and standard deviation. Normality was assessed using Shapiro–Wilk test. Univariable and multivariable linear regressions were performed to estimate associations between SF-36 domains, IES-R, and clinical/demographic variables by calculating ß (linear regression coefficient) and 95% confidence intervals (CIs). Multivariable analyses included significant variables, *p* < 0.05 from univariable analyses, considering potential collinearities and the number of subjects. No imputation was done for missing data. Statistical analyses were performed using STATA 17.

## Results

From 1 March 2020 to 1 March 2021, 1,469 pregnant women were admitted to the Department of Obstetrics and Gynecology. Of these, 62 (4.2%) met the inclusion criteria of the study. One year after hospital discharge, all patients were contacted. Twenty-nine patients did not want to participate in the study or did not answer the phone.

**Figure 1:**
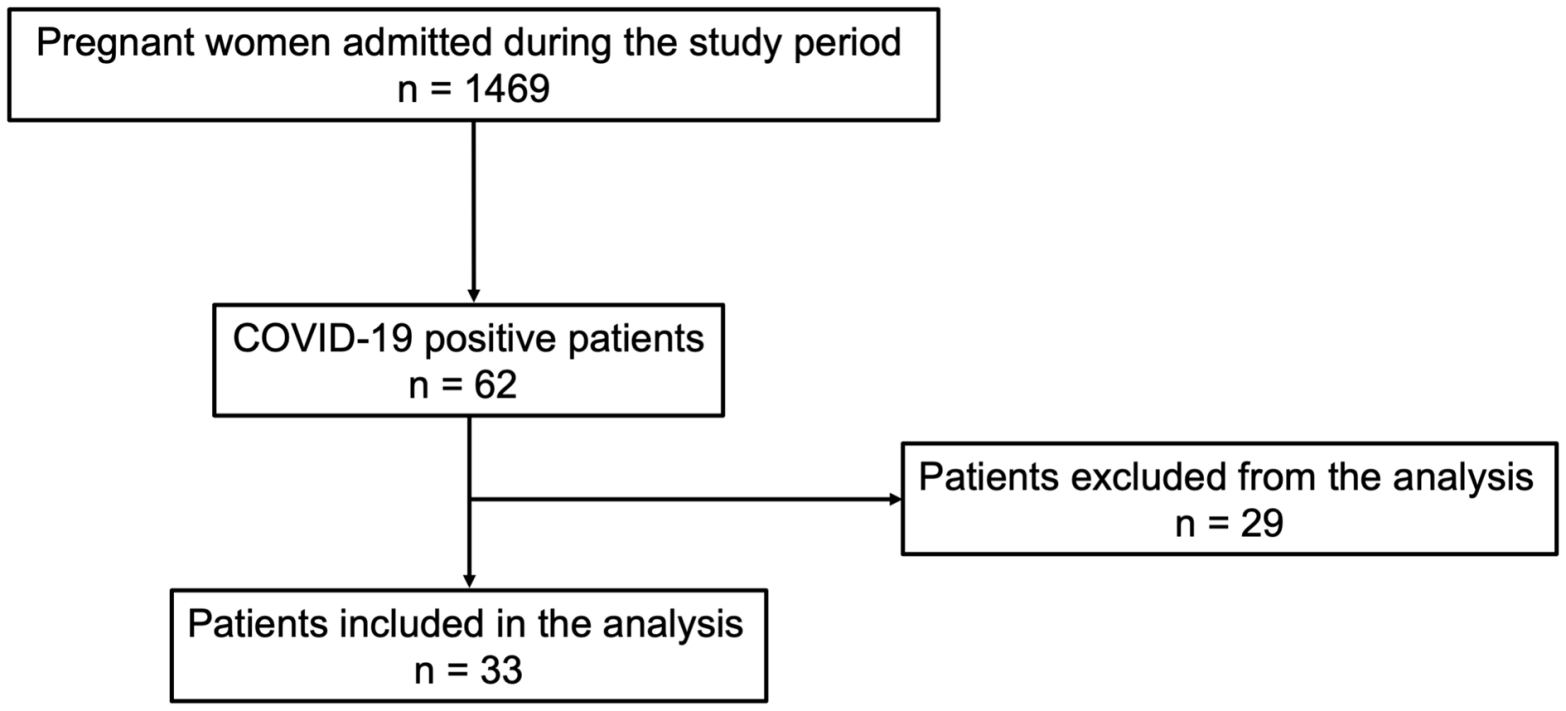
Study flow chart according to PRISMA Flowchart.

Data from 33 patients were analyzed for the final statistical analysis (Figure 1).

The mean age was 32 ± 6 years, with a median gestational age of 38 weeks [IQR 34–40].

The median body weight before COVID-19 diagnosis was 60 Kg [IQR 54.5–70], and at the 1-year follow-up was 70 Kg [IQR 64–77]. Most of the patients had a high level of education (42.4% had a degree and 36.4% a high school diploma) and were in a stable relationship (60.6% were married, and 36.4% had a regular partner). The majority (66.7%) of the patients were employed at the time of study inclusion, as shown in Table 1.

**Table 1:** Baseline characteristics of COVID-19 positive pregnant women.

	*N* = 33
Age, mean ± SD	32 ± 6
Gestational age, median [IQR]	38 [34–40]
Body weight before-COVID, median [IQR]	60 [54.5–70]
Body weight at follow-up, kg, median [IQR]	70 [64–77]
**Education, *n* (%)**	
None	1 (3.0)
Elementary School Diploma	0 (0)
Junior High School Diploma	6 (18.2)
High School Diploma	12 (36.4)
Degree	14 (42.4)
**Marital status, *n* (%)**	
Married	20 (60.6)
Stable partnership	12 (36.4)
Divorced	1 (3.0)
**Occupation, *n* (%)**	
Unoccupied	5 (15.2)
Employed	20 (60.6)
Precarious employment	2 (6.1)
Does not work	6 (18.2)
**Cardiovascular disease, *n* (%)**	
None	32 (97.0)
Hypertension	1 (3.0)
**Lung disease, *n* (%)**	
None	32 (97.0)
Asthma	1 (3.0)
Kidney disease, *n* (%)	2 (6.1)
Liver disease, *n* (%)	1 (3.0)

Only a small percentage of the women had a previous medical history (see Table 1).

The median length of hospital stay (LOS_HOSP_) was 5 days [IQR 4–7]. Steroids were administrated to 6 women (18.2%) for a median of 8 days [IQR 6–9]. In all, 15.2% of patients required oxygen therapy through noninvasive respiratory support (with high flow nasal cannula) for a median of 9 days [IQR 6–12]; 1 (3%) patient was admitted to ICU and treated with full face noninvasive pressure support ventilation (see Table 2). The majority of patients (57.6%) had natural delivery, while less than one in three (27%) had cesarean section as reported in Table 2.

**Table 2:** Main hospital stay data.

	*N* = 33
LOS_HOSP_ days, median [IQR]	5 [4–7]
Steroids, *n* (%)	6 (18.2)
Duration, median [IQR]	8 [6–9]
Supplemental oxygen therapy, *n* (%)	5 (15.2)
Duration, median [IQR]	9 [6–12]
ICU admission, *n* (%)	1 (3%)
**Type of birth, *n* (%)**	
Natural	19 (57.6)
Cesarean	9 (27.3)
Operative	5 (15.1)

LOS_HOSP_, length of hospital stay; ICU, intensive care unit.

Table 3 shows the SF-36 results. Lower scores were recorded in the bodily pain dimension.

**Table 3:** HRQoL according to SF-36 results.

SF-36 item	Mean ± SD
PF	91.7 ± 16.3
RP	86.7 ± 32.8
RE	83.3 ± 33.3
BP	57.9 ± 16.2
GH	70.2 ± 16.9
VT	81.8 ± 22.3
SF	90.7 ± 16.3
MH	76.5 ± 16.5

PF, physical functioning; PR, physical role; BP, bodily pain; GH, general health; VT, vitality; SF, social functioning; RE, emotional role; MH, mental health.

**Figure 2:**
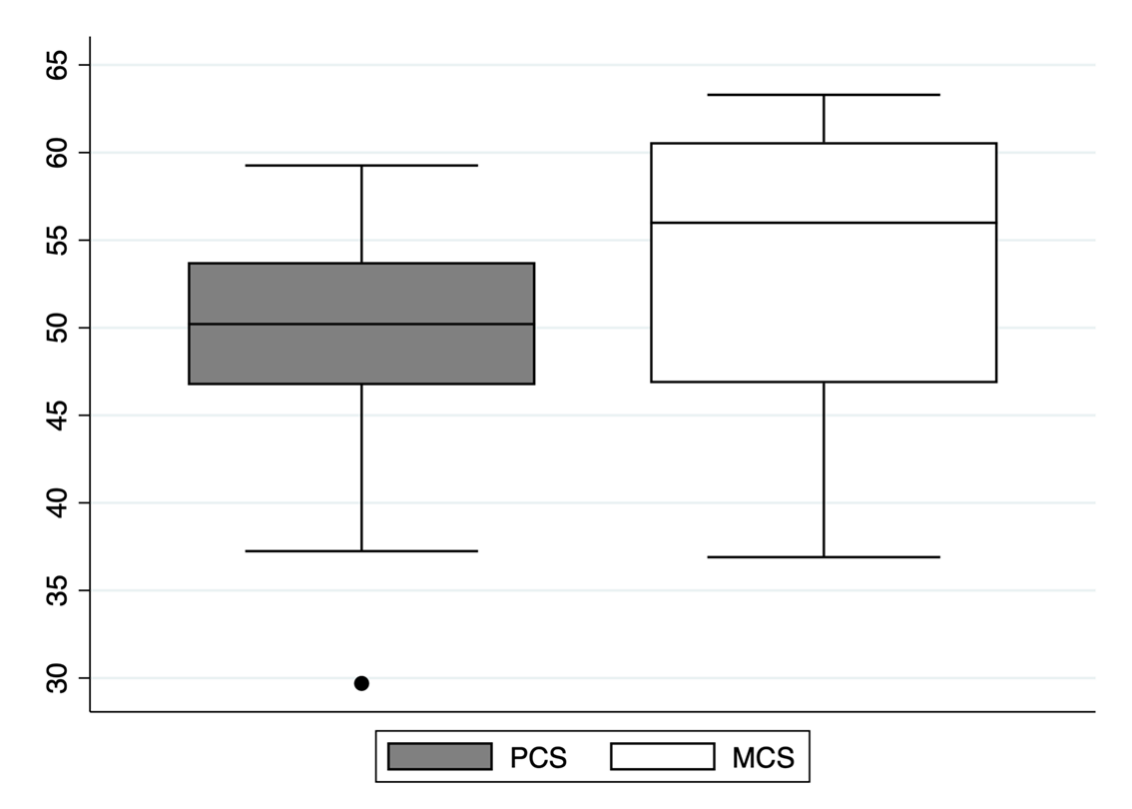
Physical (PCS) and Mental Component Summary (MCS) 1-year after hospital discharge. PCS reflects physical wellness, while MCS returns information on the global mental health condition. They are constructed using a principal component analysis based on the data of the general population of the US, standardized to obtain a mean of 50 and a standard deviation of 10.

As Figure 2 shows, the median PCS and MCS scores were 50.2 [IQR 46.7–53.7] and 56.0 [IQR 46.8–60.6]. Ten patients out of 33 (30%) tested positive for PTSD, as defined by an IES-R sum score ≥ 33. The median sum score of the entire population was 21 [IQR 10–50].

A multivariate linear regression analysis described in Supplementary Table S1 found an association between bodily pain (BP) in the SF-36 and kidney disease (β = −11.37, *p* = 0.041) and between general health (GH) and gestational age (β = 0.89, *p* = 0.021) and kidney disease (β = −13.56, *p* = 0.018). Vitality (VT) was found to be statistically significant if related to age (β = −1.20, *p* = 0.044) and gestational age (β = 1.59, *p* = 0.001), while social functioning (SF) with lung disease (β = −12.66, *p* = 0.012). Mental health (MH) results are related to gestational age (β = 0.75, *p* = 0.05). The linear regression, considering the IES-R, found a statistical result when relating intrusion with kidney disease (β = 0.81, p = 0.04), as shown in Supplementary Table S2.

## Discussion

This prospective observational study is unique and evaluated HRQoL in COVID-19 unvaccinated pregnant women 1 year after hospital discharge following childbirth.

The main findings are as follows:

1. HRQoL in unvaccinated COVID-19-positive pregnant women 1 year after hospital discharge seems less preserved in the physical than in the mental domain;
2. maternal age, gestational age, and history of cardiac-pulmonary-kidney disease significantly affected HRQoL; and
3. overall, 30% of patients tested positive for PTSD.

Interest in investigating HRQoL in pregnant women has increased over the last few years (14). It is measured through multidimensional questionnaires, with each dimension evaluated indirectly by a set of items to explore the characteristics of the investigated concept (15).

In our sample, we found lower scores in the physical status compared with the cognitive domain. The most affected score of SF-36 was bodily pain (BP), which reflects the intensity and effect of pain on everyday work both inside and outside the home (16).

Some nonclinical and clinical events condition women’s HRQoL during pregnancy, encompassing physical, psychological, and social domains (17).

For example, a recent study evaluating 50 patients previously hospitalized with COVID-19 (14 females, age 58 ± 12 years, half treated with mechanical ventilation and half treated outside intensive care settings with noninvasive ventilation) showed that diaphragm muscle weakness was still present 15 months after hospitalization for COVID-19 in patients treated without mechanical ventilation. Furthermore, diaphragm weakness was associated with dyspnea on exertion. The authors identified diaphragm muscle weakness as a correlate for persistent dyspnea in patients after COVID-19 with lung and cardiac function within normal values (18).

According to the latest WHO definition, long COVID is “…symptoms that last for at least 2 months and cannot be explained by an alternative diagnosis. Common symptoms include fatigue, shortness of breath, and cognitive dysfunction, but others generally impact everyday functioning. Symptoms may be new onset following initial recovery from an acute COVID-19 episode or persist from the initial illness. Symptoms may also fluctuate or relapse over time” (19).

In our study, we did not explicitly measure the duration of symptoms. However, we found that many women still complained of BP, less social functioning, and mental health problems 1 year after the initial infection, symptoms that are all consistent with the WHO definition and in line with previous studies on long Covid in women who are not pregnant (20).

It is notable that women have a higher risk of developing long COVID (21). Therefore, our findings are not unexpected, particularly in light of the inclusion of women from the first pandemic waves before vaccinations were available.

Several studies have found that people infected with the pre-omicron virus have a higher risk of developing long COVID, probably for several reasons related to viral factors, host response to the infection, and pre-existing immunity (natural or acquired by vaccination) (22).

In fact, in our population, none of the patients was vaccinated against COVID-19, which might have impacted the symptoms and the LOS_HOSP_ since it has been demonstrated that vaccination reduces the severity and duration of long COVID (23).

This last is also associated with psychiatric symptoms (24).

Screening in our sample determined that 30% of patients could have PTSD.

In Delanerolle et al.’s systematic review and meta-analysis that considered more than 638,000 women, the symptoms of PTSD measured with the IES and the fifth edition of the PTSD checklist for Diagnostic and Statistical Manual of Mental Disorders (PCL-5) found a prevalence of 27.93% with a 95%CI of 9.05%–86.15% (25).

Considering COVID-19 patients admitted to the hospital, a recent study that evaluated 343 ICU patients 1 year after discharge showed similar results, with 31.8% of patients testing positive for PTSD (26).

In a cross-sectional study with an online survey, Motrico et al. found a prevalence of >40% of PTSD in pregnant and postpartum women affected by COVID-19 using the PCL-5. In their conclusion, they do not point out COVID-19 as a risk factor for PTSD (27).

It is challenging to determine whether PTSD is caused by the COVID-19 pandemic or by postpartum depression.

Having problems during childbirth due to the pandemic, the infection of family members/loved ones with COVID-19, and the follow-up of the national situation related to COVID-19 may all have significantly affected the PTSD symptoms of pregnant women (28).

However, it is essential to consider that COVID-19 should be regarded as an additional stressor factor that may contribute to an increase in the risk of PTSD. Furthermore, in an Italian study conducted during the first waves of COVID-19 emerged that stress related to the pandemic co-occurred with pregnancy-specific stress. Both types of stress were potent predictors of poorer mental health and development of anxiety, depressive, and obsessive-compulsive symptoms (29).

For these reasons, prolonged and repeated follow-up visits are highly warranted to reevaluate physical improvements after hospitalization for COVID-19 among pregnant women.

As a general matter, many factors could influence physical performance after childbirth: fatigue, dyspnea, back pain, itching in the cesarean incision, and perineal pain are commonly reported problems for some weeks after delivery (30, 31). During the COVID-19 pandemic, the social restrictions and limited access to the hospital to limit the viral spread and new possible contagions also reduced opportunities for women to receive medical consultation to explain or solve their problems (32).

The neurological effects of COVID-19 also need to be considered (33), since, despite the high scores reported for PF, the presence of lower scores for BP point to a relationship between physical and mental feelings that the patients may have interpreted as pain.

Therefore, it is crucial to determine if some factors could be related to better or worse HRQoL. Age was a significant factor in worsening BP, VT, and MH.

There is contrasting evidence about older age as a factor that lowers HRQoL (34). Park et al. recently demonstrated that women of an early age at first childbirth tended to have lower HRQoL because they experienced more deliveries (35). Conversely, Martínez-Galiano et al. found that maternal age was not associated with HRQoL after birth in a large sample of women (36).

Similar to our results, Liu et al. clearly showed how the mother’s age at delivery significantly associates with HRQoL as a decreasing factor (34).

Increasing gestational age was a protective factor against lower general health, vitality, and mental health scores. Estebsari et al. also reported this finding in a large cohort of Iranian women (37).

We argue that overcoming the most delicate phase of pregnancy, when the fetus can be lost more quickly, can produce maternal well-being, reflecting a better HRQoL.

Conversely, a history of cardiac, kidney, or pulmonary disease decreases HRQoL, as women in this study reported thanks to SF-36.

These women are likely faced with more problems during pregnancy. Moreover, they probably needed more and more medical consultations. However, during the COVID-19 pandemic, they likely encountered many impairments in hospital access, which impacted HRQoL. It is interesting to note that the need for oxygen therapy during hospital stay reduced SF score.

The modality of oxygen supplementation has long been debated to find the best way for respiratory support (38).

In this regard, lung ultrasound as a screening tool to decide whether pregnant patients with COVID-19 can return home or should be admitted to the hospital for eventual oxygen supplementation could be valuable at the first medical contact (39).

Although not specifically designed to understand the burden of long COVID (or post-COVID condition), our study offers clues to hypothesize that this syndrome can also affect young pregnant women (40).

This study has some limitations. First, some women were lost at the follow-up or wanted to avoid participating in the study and thus did not give us data regarding their status, which contributed to the risk of excluding patients with lower HRQoL.

Second, the questionnaires were self-administrated, and even if they were easy to interpret, they might not have yielded completely accurate data.

Third, the generalizability of these data should be approached with caution. Evidence suggests that HRQoL also depends on the region where women live, especially due to the socioeconomic disparity of study participants (41).

Fourth, we did not compare our results to non-infected pregnant women self-reported HRQoL. However, we took a snapshot on this, and describing the HRQoL in a particular selected population could help to understand the disease, adding evidence to the literature.

Finally, the study has a limited sample size and it would be interesting to analyze data of a larger number of pregnant women.

## Conclusion

One year after hospital discharge in COVID-19 pregnant women, some physical impairments reducing HRQoL are still present. Previous medical history significantly affected HRQoL. Moreover, one out of three patients tested positive for PTSD.

This category of patients is particularly prone to physical and mental disorders and thus may need extensive, continuous follow-up to speed up the recovery phase after viral infection and childbirth.

## Data availability statement

The raw data supporting the conclusions of this article will be made available by the authors, without undue reservation.

## Ethics statement

The studies involving humans were approved by Comitato Etico Unico Regionale FVG. The studies were conducted in accordance with the local legislation and institutional requirements. The participants provided their written informed consent to participate in this study.

## Author contributions

LV, CD, and LD were responsible for conceptualization, data curation, methodology, data analysis, interpretation, writing original draft, and review and editing. AS, FM, MI, MM, SR, and GV were responsible for data curation, methodology, data analysis, interpretation, writing original draft, and review and editing. MG and TB were responsible for data curation, data analysis, interpretation, writing original draft, and review and editing. SM was responsible for methodology, data analysis, interpretation, writing original draft, and review and editing. All authors contributed to the article and approved the submitted version.

## Conflict of interest

The authors declare that the research was conducted in the absence of any commercial or financial relationships that could be construed as a potential conflict of interest.

## Publisher’s note

All claims expressed in this article are solely those of the authors and do not necessarily represent those of their affiliated organizations, or those of the publisher, the editors and the reviewers. Any product that may be evaluated in this article, or claim that may be made by its manufacturer, is not guaranteed or endorsed by the publisher.
