# Supplementary material for: Quality of life 1 year after hospital discharge in unvaccinated pregnant women with COVID-19 respiratory symptoms: a prospective observational study (ODISSEA-PINK study)
Source: Front Med (Lausanne). 2023 Sep 6;10:1225648. doi: 10.3389/fmed.2023.1225648 (PMC10516577; doi:10.3389/fmed.2023.1225648)
Supplement: Supplementary file 1 [file Table_1.doc]

**Table S1. Baseline, hospital and therapy data relationship with SF-36 parameters.**

|  | **Univariable analysis** | | |
| --- | --- | --- | --- |
| **Physical Function** | **ß** | **95% CI** | **p-value** |
| **Age** | 0.31 | -0.75,1.36 | 0.557 |
| **GA at hospital admission** | 0.08 | -0.76,0.92 | 0.846 |
| **Weight before COVID infection** | -0.03 | -0.44,0.39 | 0.903 |
| **Weight at follow-up** | -0.11 | -0.84,0.62 | 0.743 |
| **Cardiac disease** | -17.19 | -50.97,16.59 | 0.307 |
| **Pulmonary disease** | -2.29 | -13.72,9.13 | 0.685 |
| **Kidney disease** | -2.22 | -14.53,10.10 | 0.716 |
| **Liver disease** | -2.29 | -13.72,9.13 | 0.685 |
| **LOS_HOSP_** | -0.40 | -2.17,1.37 | 0.648 |
| **Steroid use** | 3.06 | -12.18,18.29 | 0.685 |
| **Oxygen therapy** | -1.96 | -18.38,14.45 | 0.809 |
| **Type of birth**  **Natural**  **Cesarean**  **Operative** | 1  -10.09  6.58 | -23.16,2.98  -9.65,22.81 | 0.125  0.414 |
| **Number of previous pregnancies** | 2.64 | -4.49,9.78 | 0.456 |

|  | **Univariable analysis** | | |
| --- | --- | --- | --- |
| **Role Physical** | **ß** | **95% CI** | **p-value** |
| **Age** | -0.03 | -2.15,2.09 | 0.974 |
| **GA at hospital admission** | 0.28 | -1.39,1.96 | 0.732 |
| **Weight before COVID infection** | -0.15 | -0.94,0.63 | 0.695 |
| **Weight at follow-up** | 13.67age | -55.10,82.44 | 0.688 |
| **Cardiac disease** | -12.63 | -35.14,9.88 | 0.261 |
| **Pulmonary disease** | 7.06 | -17.58,31.69 | 0.563 |
| **Kidney disease** | 4.56 | -18.37,27.48 | 0.688 |
| **Liver disease** | -1.18 | -4.72,2.36 | 0.502 |
| **LOS_HOSP_** | -1.62 | -32.26,29.02 | 0.915 |
| **Steroid use** | -5 | -37.91,27.91 | 0.759 |
| **Type of birth**  **Natural**  **Cesarean**  **Operative** | 1  6.65  17.76 | -20.78,34.08  -16.31,51.83 | 0.624  0.295 |
| **Number of previous pregnancies** | 4.62 | -9.74,18.97 | 0.517 |

|  | **Univariable analysis** | | |
| --- | --- | --- | --- |
| **Role Emotional** | **ß** | **95% CI** | **p-value** |
| **Age** | -0.72 | -2.86,1.43 | 0.501 |
| **GA at hospital admission** | -0.14 | -1.85,1.56 | 0.866 |
| **Weight before COVID infection** | 0.16 | -0.65,0.98 | 0.682 |
| **Weight at follow-up** | -0.82 | -2.12,0.48 | 0.194 |
| **Cardiac disease** | 17.19 | -52.67,87.05 | 0.619 |
| **Pulmonary disease** | 5.73 | -17.56,29.02 | 0.619 |
| **Kidney disease** | 8.87 | -16.11,33.86 | 0.474 |
| **Liver disease** | 5.73 | -17.56,29.02 | 0.619 |
| **LOS_HOSP_** | 0.41 | -3.21,4.04 | 0.817 |
| **Steroid use** | 0.01 | -31.17,31.17 | 1.000 |
| **Oxygen therapy** | 3.93 | -29.58,37.43 | 0.813 |
| **Type of birth**  **Natural**  **Cesarean**  **Operative** | 1  -9.26  16.67 | -36.83,18.31  -17.58,50.91 | 0.498  0.328 |
| **Number of previous pregnancies** | -3.78 | -18.42,10.86 | 0.602 |

|  | **Univariable analysis** | | | **Multivariable analysis** | | |
| --- | --- | --- | --- | --- | --- | --- |
| **Bodily Pain** | **ß** | **95% CI** | **p-value** | **ß** | **95% CI** | **p-value** |
| **Age** | -1.11 | -2.07,-0.15 | **0.026** | -0.91 | -1.85,0.02 | 0.054 |
| **GA at hospital admission** | 0.72 | -0.06,1.51 | 0.070 |  |  |  |
| **Weight before COVID infection** | -0.24 | -0.67,0.19 | 0.264 |  |  |  |
| **Weight at follow-up** | -0.38 | -1.18,0.41 | 0.314 |  |  |  |
| **Cardiac disease** | -28.75 | -61.07,3.57 | 0.079 |  |  |  |
| **Pulmonary disease** | -4.43 | -15.64,6.79 | 0.427 |  |  |  |
| **Kidney disease** | -13.51 | -24.67,-2.34 | **0.019** | -11.37 | -22.27,-0.49 | **0.041** |
| **Liver disease** | -0.99 | -12.32,10.34 | 0.860 |  |  |  |
| **LOS_HOSP_** | -0.39 | -2.14,1.36 | 0.653 |  |  |  |
| **Steroid use** | -3.52 | -18.57,11.54 | 0.637 |  |  |  |
| **Oxygen therapy** | -11.64 | -27.33,4.04 | 0.140 |  |  |  |
| **Type of birth**  **Natural**  **Cesarean**  **Operative** | 1  -4.06  14.16 | -16.90,8.77  -1.78,30.10 | 0.523  0.080 |  |  |  |
| **Number of previous pregnancies** | -3.16 | -10.19,3.87 | 0.367 |  |  |  |

|  | **Univariable analysis** | | | **Multivariable analysis** | | |
| --- | --- | --- | --- | --- | --- | --- |
| **General Health** | **ß** | **95% CI** | **p-value** | **ß** | **95% CI** | **p-value** |
| **Age** | -0.91 | -1.95,0.14 | 0.086 |  |  |  |
| **GA at hospital admission** | 0.85 | 0.04,1.65 | **0.041** | 0.89 | 0.14,1.64 | **0.021** |
| **Weight before COVID infection** | 0.01 | -0.44,0.47 | 0.956 |  |  |  |
| **Weight at follow-up** | -0.17 | -1.01,0.67 | 0.661 |  |  |  |
| **Cardiac disease** | -14.63 | -49.75,20.50 | 0.402 |  |  |  |
| **Pulmonary disease** | 0.63 | -11.22,12.47 | 0.915 |  |  |  |
| **Kidney disease** | -12.87 | -24.73,-1.01 | **0.034** | -13.56 | -24.60,-25.11 | **0.018** |
| **Liver disease** | -0.75 | -12.59,11.09 | 0.898 |  |  |  |
| **LOS_HOSP_** | -0.30 | -2.14,1.53 | 0.739 |  |  |  |
| **Steroid use** | -1.85 | -17.63,13.92 | 0.812 |  |  |  |
| **Oxygen therapy** | -1.63 | -18.60,15.35 | 0.846 |  |  |  |
| **Type of birth**  **Natural**  **Cesarean**  **Operative** | 1  -13.03  1.73 | -26.47,0.41  -14.97,18.42 | 0.057  0.834 |  |  |  |
| **Number of previous pregnancies** | -1.88 | -9.30,5.54 | 0.609 |  |  |  |

|  | **Univariable analysis** | | | **Multivariable analysis** | | |
| --- | --- | --- | --- | --- | --- | --- |
| **Vitality** | **ß** | **95% CI** | **p-value** | **ß** | **95% CI** | **p-value** |
| **Age** | -1.37 | -2.72,-0.01 | **0.048** | -1.20 | -2.36,-0.04 | **0.044** |
| **GA at hospital admission** | 1.67 | 0.71,2.64 | **0.001** | 1.59 | 0.67,2.51 | **0.001** |
| **Weight before COVID infection** | -0.44 | -1.01,0.13 | 0.125 |  |  |  |
| **Weight at follow-up** | -0.28 | -1.03,0.48 | 0.440 |  |  |  |
| **Cardiac disease** | -32.81 | -78.26,12.63 | 0.151 |  |  |  |
| **Pulmonary disease** | -10.94 | -26.08,4.21 | 0.151 |  |  |  |
| **Kidney disease** | -6.96 | -23.64,9.73 | 0.402 |  |  |  |
| **Liver disease** | 1.95 | -13.70,17.60 | 0.801 |  |  |  |
| **LOS_HOSP_** | -0.87 | -3.28,1.54 | 0.465 |  |  |  |
| **Steroid use** | -8.33 | -29.0,12.33 | 0.417 |  |  |  |
| **Oxygen therapy** | -13.93 | -35.81,7.96 | 0.204 |  |  |  |
| **Type of birth**  **Natural**  **Cesarean**  **Operative** | 1  3.73  7.89 | -15.17,22.63  -15.58,31.37 | 0.690  0.497 |  |  |  |
| **Number of previous pregnancies** | -2.47 | -12.28,7.34 | 0.611 |  |  | |

|  | **Univariable analysis** | | | **Multivariable analysis** | | |
| --- | --- | --- | --- | --- | --- | --- |
| **Social Functioning** | **ß** | **95% CI** | **p-value** | **ß** | **95% CI** | **p-value** |
| **Age** | -0.85 | -1.86,0.16 | 0.095 |  |  |  |
| **GA at hospital admission** | 0.23 | -0.60,1.06 | 0.580 |  |  |  |
| **Weight before COVID infection** | -0.45 | -0.87,-0.04 | **0.031** |  |  |  |
| **Weight at follow-up** | -0.37 | -1.11,0.37 | 0.299 |  |  |  |
| **Cardiac disease** | -36.80 | -68.42,-5.17 | **0.024** | -37.98 | -66.91,-9.06 | **0.012** |
| **Pulmonary disease** | -12.27 | -22.81,-1.72 | **0.024** | -12.66 | -22.30,-3.02 | **0.012** |
| **Kidney disease** | -7.02 | -19.09,5.06 | 0.245 |  |  |  |
| **Liver disease** | -7.97 | -19.05,3.11 | 0.153 |  |  |  |
| **LOS_HOSP_** | 0.19 | -1.58,1.97 | 0.827 |  |  |  |
| **Steroid use** | -8.47 | -23.43,6.49 | 0.257 |  |  |  |
| **Oxygen therapy** | -17.30 | -32.47,-2.14 | **0.027** |  |  |  |
| **Type of birth**  **Natural**  **Cesarean**  **Operative** | 1  7.56  13.42 | -5.63,20.81  -3.00,29.84 | 0.250  0.105 |  |  |  |
| **Number of previous pregnancies** | -3.99 | -11.04,3.07 | 0.258 |  |  | |

|  | **Univariable analysis** | | | **Multivariable analysis** | | |
| --- | --- | --- | --- | --- | --- | --- |
| **Mental Health** | **ß** | **95% CI** | **p-value** | **ß** | **95% CI** | **p-value** |
| **Age** | -1.09 | -2.08,-0.10 | **0.032** | -1.01 | -1.96,-0.07 | **0.037** |
| **GA at hospital admission** | 0.82 | 0.03,1.61 | **0.043** | 0.75 | -0.01,1.50 | 0.050 |
| **Weight before COVID infection** | -0.24 | -0.67,0.20 | 0.275 |  |  |  |
| **Weight at follow-up** | -0.30 | -1.05,0.44 | 0.390 |  |  |  |
| **Cardiac disease** | -37.66 | -69.55,-5.77 | **0.022** |  |  |  |
| **Pulmonary disease** | -7.40 | -18.66,3.86 | 0.190 |  |  |  |
| **Kidney disease** | -14.11 | -25.47,-2.75 | **0.017** |  |  |  |
| **Liver disease** | -0.52 | -12.10,11.06 | 0.928 |  |  |  |
| **LOS_HOSP_** | -0.51 | -2.30,1.28 | 0.564 |  |  |  |
| **Steroid use** | -8.98 | -24.07,6.11 | 0.234 |  |  |  |
| **Oxygen therapy** | -10.04 | -26.24,6.16 | 0.216 |  |  |  |
| **Type of birth**  **Natural**  **Cesarean**  **Operative** | 1  -13.13  9.32 | -25.66,-0.60  -6.24,24.88 | **0.041**  0.231 |  |  |  |
| **Number of previous pregnancies** | -4.70 | -11.78,2.37 | 0.185 |  |  | |

**Table S2. IES-R and baseline, clinical and hospital data effects.**

|  | **Univariable analysis** | | |
| --- | --- | --- | --- |
| **Avoidance** | **ß** | **95% CI** | **p-value** |
| **Age** | 0.01 | -0.05,0.07 | 0.696 |
| **GA at hospital admission** | -0.02 | -0.07,0.02 | 0.340 |
| **Weight before COVID infection** | -0.02 | -0.04,0.01 | 0.087 |
| **Weight at follow-up** | -0.01 | -0.04,0.04 | 0.972 |
| **Cardiac disease** | 0.80 | -1.09,2.68 | 0.395 |
| **Pulmonary disease** | -0.29 | -0.92,0.33 | 0.344 |
| **Kidney disease** | 0.48 | -0.18,1.14 | 0.151 |
| **Liver disease** | 0.09 | -0.54,0.73 | 0.765 |
| **LOS_HOSP_** | -0.04 | -0.14,0.06 | 0.434 |
| **Steroid use** | -0.38 | -1.22,0.45 | 0.356 |
| **Oxygen therapy** | -0.01 | -0.92,0.90 | 0.988 |
| **Type of birth**  **Natural**  **Cesarean**  **Operative** | 1  0.22  0.21 | -0.54,0.99  -0.74,1.17 | 0.556  0.650 |
| **Number of previous pregnancies** | -0.20 | -0.59,0.19 | 0.308 |

|  | **Univariable analysis** | | |
| --- | --- | --- | --- |
| **Intrusion** | **ß** | **95% CI** | **p-value** |
| **Age** | 0.04 | -0.03,0.11 | 0.277 |
| **GA at hospital admission** | -0.01 | -0.07,0.04 | 0.624 |
| **Weight before COVID infection** | -0.02 | -0.05,0.01 | 0.138 |
| **Weight at follow-up** | -0.01 | -0.07,0.04 | 0.586 |
| **Cardiac disease** | 1.57 | -0.66,3.80 | 0.160 |
| **Pulmonary disease** | -0.42 | -1.17,0.33 | 0.262 |
| **Kidney disease** | 0.81 | 0.04,1.58 | **0.040** |
| **Liver disease** | 0.57 | -0.17,1.31 | 0.126 |
| **LOS_HOSP_** | 0.03 | -0.09,0.15 | 0.640 |
| **Steroid use** | -0.20 | -1.22,0.82 | 0.696 |
| **Oxygen therapy** | 0.39 | -0.71,1.48 | 0.477 |
| **Type of birth**  **Natural**  **Cesarean**  **Operative** | 1  0.67  0.45 | -0.23,1.56  -0.66,1.57 | 0.140  0.415 |
| **Number of previous pregnancies** | -0.20 | -0.68,0.27 | 0.389 |

|  | **Univariable analysis** | | |
| --- | --- | --- | --- |
| **Iperarousal** | **ß** | **95% CI** | **p-value** |
| **Age** | 0.02 | -0.05,0.08 | 0.632 |
| **GA at hospital admission** | -0.01 | -0.05,0.05 | 0.979 |
| **Weight before COVID infection** | -0.02 | -0.05,0.01 | 0.074 |
| **Weight at follow-up** | -0.01 | -0.06,0.05 | 0.832 |
| **Cardiac disease** | 1.40 | -0.77,3.57 | 0.197 |
| **Pulmonary disease** | -0.39 | -1.12,0.34 | 0.280 |
| **Kidney disease** | 0.72 | -0.03,1.48 | 0.060 |
| **Liver disease** | 0.58 | -0.13,1.29 | 0.106 |
| **LOS_HOSP_** | -0.02 | -0.13,0.10 | 0.731 |
| **Steroid use** | -0.44 | -1.42,0.53 | 0.361 |
| **Oxygen therapy** | 0.19 | -0.88,1.25 | 0.723 |
| **Type of birth**  **Natural**  **Cesarean**  **Operative** | 1  0.40  0.43 | -0.48,1.29  -0.67,1.53 | 0.359  0.427 |
| **Number of previous pregnancies** | -0.22 | -0.68,0.24 | 0.338 |
